# Emergency Department utilization in Switzerland: Comparing Swiss natives with first- and second-generation immigrants

**DOI:** 10.1016/j.jmh.2025.100388

**Published:** 2026-01-02

**Authors:** Ludovica Alesci, Igor Francetic

**Affiliations:** a Competence Centre for Healthcare Practices and Policies, University of Applied Sciences and Arts of Southern Switzerland (SUPSI), Switzerland; b Health Organisation, Policy and Economics, University of Manchester, UK

**Keywords:** Inequalities, Health care, Migration, Health equity, Immigrants, Emergency Department

## Abstract

**Introduction:**

Differences in Emergency Department (ED) utilization between immigrant and native populations may reflect inequalities in health status and access to care. This study compares ED use between Swiss natives and first- and second-generation immigrants in Switzerland.

**Methods:**

We used pooled data from the Swiss Health Survey 2017 and 2022 (N = 16,183). Logistic regression models were estimated and reported as average marginal effects (AMEs). Models were progressively adjusted for sociodemographic characteristics, health status, health behaviors, and healthcare-use variables.

**Results:**

In unadjusted models, first-generation immigrants showed a higher probability of ED use (AME = 0.025, SE = 0.004, p<0.001; 95% CI: 0.017–0.033), but this association disappeared after adjusting for health status (AME = 0.007, SE = 0.006, p = 0.25). For second-generation immigrants, the association remained significant after adjustments (Main model: AME = 0.030, SE = 0.010, p = 0.01; 95% CI: 0.011–0.049) and slightly attenuated when accounting for healthcare-use patterns (AME = 0.022, SE = 0.011, p = 0.06; 95% CI: 0.000–0.042). An alternative analysis based on Oaxaca–Blinder decompositions confirmed that differences between Swiss natives and first-generation immigrants are mainly explained by health status, whereas differences with second-generation immigrants remain largely unexplained.

**Conclusions:**

First-generation immigrants do not differ from Swiss natives in ED use once differences in health status are taken into account. Second-generation immigrants (particularly women and individuals from Eastern and South-Eastern Europe) exhibit a persistently higher probability of ED use, partly explained by higher engagement with other healthcare services. These findings highlight the need for targeted interventions to improve equitable access and continuity of care among immigrant populations.

## Highlights

• Second-generation immigrants use emergency care more than native Swiss.
• Health explains differences between Swiss natives and First-generation immigrants.
• Differences are concentrated among women and people from Eastern and Southern Europe.

## 1 Introduction

In Europe and other high-income regions, migration is a key driver of demographic growth. In Switzerland, 40% of the permanent resident population aged 15 and over had a migration background in 2023. Of these, almost 80% were first-generation immigrants, while the remaining 20% were second-generation immigrants born in Switzerland (FSO, 2023).^1^ Given this substantial share of the population, it is essential to ensure that healthcare services are accessible and effective for these groups. However, disparities in healthcare access and utilization between native Swiss residents and immigrants remain a significant concern as they may have important implications for public health, social equity, and the overall efficiency of the health system.

The Swiss Federal Government has made equitable access to healthcare for all residents a priority, regardless of background. However, research shows that individuals with a migration background are less likely to access some preventive healthcare services, such as cancer screening programs (Fontana and Bischoff, 2008). In addition, some studies have reported that older immigrants may experience higher hospitalization rates and more frequent physician visits compared to native-born populations (Solé-Auró et al., 2012, Jaeger et al., 2012).

Immigrants are often healthier compared to the population of their host countries, a phenomenon known as the *”healthy immigrant effect”* (Neuman, 2014). Immigrants typically exhibit better health upon arrival, but over time, their health status tends to decline and converge with that of native-born individuals (Dunn and Dyck, 2000, McDonald and Kennedy, 2004, Newbold, 2005, Lu and Qin, 2014). For example, those who migrated for employment, family, and study reasons report better health outcomes than natives, while those who migrated to seek asylum report worse health outcomes than natives (Giuntella et al., 2018). The initial health advantage observed among immigrants is not only attributed to positive selection, where people with poor health are less likely to migrate successfully (Ichou and Wallace, 2019, Kennedy et al., 2015, Giuntella and Lonsky, 2022). It also comes from the fact that migration decisions often align with higher levels of socioeconomic status, education, and access to resources that promote healthier behaviors prior to migration. However, over time, this health advantage tends to erode as immigrants adopt the host country’s lifestyle and dietary habits or encounter the stress and challenges associated with integration (Kennedy et al., 2015, Giuntella and Lonsky, 2022).

Despite these initial health benefits, research consistently reveals significant disparities in healthcare access and utilization between immigrant and native-born populations (Tzogiou et al., 2021, Muennig and Fahs, 2002, Norredam et al., 2007, Nielsen et al., 2012). Although not all immigrants experience the same barriers, some may face additional difficulties related to limited awareness of their entitlements (Szczepura, 2005), fear of being reported (Rechel et al., 2012), and discriminatory practices (Tzogiou et al., 2021). While language and administrative obstacles represent socially unjust barriers, cultural differences may instead reflect acceptable variations; however, they can still pose challenges in the delivery of healthcare (Terui, 2017, Tzogiou et al., 2021). Specific groups, such as immigrant women, face additional obstacles related not only to linguistic and cultural barriers but also to gendered expectations and roles. Some of these challenges, such as limited access to interpretation and translation services, affect communication with healthcare providers and can result in misunderstandings, incomplete assessments, and missed follow-up appointments (Gil-Salmerón et al., 2021, Lebano et al., 2020). When combined with cultural expectations and gendered responsibilities, these factors may disproportionately impact immigrant women, leading to further barriers in accessing care. Communication and information gaps, along with issues such as limited health literacy and culturally insensitive services, contribute to increasing health vulnerabilities. These barriers, combined with the perception among immigrants of unmet healthcare needs due to challenges in accessing adequate or equitable services, highlight the need for improved healthcare accessibility and support tailored to immigrants’ diverse backgrounds and experiences (Lebano et al., 2020).

Emergency Department (ED) plays a crucial role as the front line of healthcare, providing essential services to diverse groups in urgent circumstances. This unique healthcare setting bridges outpatient (ambulatory) care and inpatient (hospital-based) care. Some evidence suggests that immigrants use healthcare services, including ED, differently compared to native populations. These differences in ED usage can often be influenced by factors such as language barriers, cultural differences, socioeconomic status, and access to primary care, raising important questions about how to optimize healthcare services for diverse populations (Credé et al., 2018, Sarría-Santamera et al., 2016). Previous research has shown that migrant populations tend to use EDs more frequently and differently than native-born individuals. In particular, migrants are more likely to present with low-acuity conditions and to seek care during unsocial hours, patterns that suggest barriers to accessing primary healthcare may contribute to their higher ED utilization (Credé et al., 2018).

In Switzerland, immigrants tend to rely more on ED and acute care services, often seeking care during unsocial hours or perceiving ED services as the primary point of access for immigrants, leading to disruptions in continuity of care. Variations in healthcare utilization are also noted depending on the immigrants’ country of origin and their level of social integration. Furthermore, certain conditions, such as accidents, infectious diseases, and mental health issues, are more commonly treated among immigrants compared to chronic conditions. However, disparities in hospital use also exist, as immigrants may use public hospital services less frequently than native populations. These patterns of healthcare use are influenced by a variety of interconnected factors, including socio-economic circumstances, health status, and the healthcare system’s capacity to address the specific needs of immigrants (Lebano et al., 2020).

Specifically, immigrants are more likely to use EDs for non-urgent conditions, underutilize preventive care, and experience delays in accessing primary care. These issues can result in late diagnoses, fragmented care, and potentially worse health outcomes, representing a significant public health concern. From a social justice perspective, this is particularly troubling, as it reflects the barriers immigrants face in obtaining timely and appropriate healthcare. Examples of barriers include organizational and administrative challenges (Szczepura, 2005), language difficulties and poor health literacy (Terui, 2017), lack of knowledge of the healthcare system (Tzogiou et al., 2021), and cultural differences (Costa-Font and Sato, 2025, Rechel et al., 2012), all of which can result in underuse or inadequate care.

Given these patterns, this article aims to answer the following key research questions: (1) Do immigrants and Swiss nationals show significant differences in their use of healthcare services, particularly Emergency Departments? (2) What are the most likely factors underpinning these differences?

## 2 Institutional background

Switzerland’s healthcare system ensures access to medical services for all residents, including immigrants, as mandated by the Federal Health Insurance Act (KVG/LAMal) and supported by the Swiss Federal Constitution, which emphasizes equality and non-discrimination. Since 1996, all residents must obtain mandatory health insurance (MHI) coverage from competing insurers within their canton,^2^ with options varying in premiums, deductibles, and restrictions. Residents have the freedom to choose their insurer within their canton of residence, with multiple plans available from each provider, which can differ significantly in terms of premiums, deductibles, and doctor or hospital restrictions (De Pietro et al., 2015). In addition to MHI, individuals can opt for supplementary and complementary voluntary health insurance. Statutory health insurance covers a broad range of services, including general practitioner and specialist consultations, numerous pharmaceuticals, medical devices, home care, physiotherapy (when prescribed), and selected preventive measures, such as specific vaccinations, general health assessments, and screenings for early disease detection in certain at-risk groups (Camenzind, 2015).

To address the issue of steadily rising healthcare costs, Swiss law provides a system of financial subsidies for healthcare insurance, aimed at ensuring that lower-income individuals, including many immigrants, can access necessary healthcare services (Gerritzen et al., 2014). These subsidies are income-based, but immigrant populations with lower socioeconomic status can face challenges in navigating the bureaucratic processes required to obtain financial aid. In 2019, Switzerland enacted the Federal Act on Foreign Nationals and Integration, which underscores the importance of fostering the social and economic integration of immigrants, including their access to healthcare services. The provision of ED services is managed by the cantons, which primarily assign these responsibilities to regional medical associations and public hospitals. Prehospital care is handled by practicing physicians and emergency rescue services, while public hospital ED take on a significant portion of the responsibilities at the hospital level (Osterwalder, 1998).

Upon arriving at the ED, patients are first triaged to assess the severity of their condition and assign the appropriate level of urgency. This triage process determines how quickly a patient will be seen and by which service. Once triaged, patients are typically evaluated by medical residents, who handle the majority of initial consultations, although attending physicians are sometimes involved. The training and experience of residents vary across EDs, with some requiring no prior postgraduate experience and others requiring months of training. Residents’ rotations in the ED are usually brief, with durations varying depending on the size of the hospital (Sanchez et al., 2013).

The availability of advanced life support, such as cardiac, trauma, or pediatric life support, is a key factor in patient care but differs significantly between facilities. Larger EDs are generally better equipped with physicians trained in these areas and can respond more quickly to critical cases. Access to specialists also varies; smaller EDs often have limited or no immediate availability of specialists in radiology, pediatrics, psychiatry, or neurosurgery. These disparities mean that the process of entering an ED and receiving care can differ substantially depending on the size and resources of the facility, which may influence both the quality and speed of care provided to patients (Sanchez et al., 2013).

## 3 Conceptual framework

Disparities in ED use between immigrants and Swiss nationals can be due to individual, provider, and systemic factors. Our study is grounded in the Health Equity theory, which asserts that everyone should have a fair opportunity to achieve their highest level of health (Braveman et al., 2018). This principle serves as the foundation for the two theoretical models integrated into this research: the *Social Determinants of Health* (SDoH) framework and *Andersen’s Behavioral Model*.

The SDoH framework expands the understanding of health by considering the societal conditions that influence health outcomes. It argues that health is not solely shaped by healthcare services but also by social, economic, and environmental factors in which individuals live (Marmot, 2005). These factors include socioeconomic status, education, living conditions, and employment, all of which are often influenced by structural inequalities. Immigrants, who are frequently placed in disadvantaged positions within social hierarchies, can experience poorer health outcomes as a result (Castañeda et al., 2015). While immigrants represent a highly heterogeneous population, the SDoH framework suggests that those with lower socioeconomic status may face barriers to preventive healthcare, resulting in greater reliance on ED services (Marmot and Allen, 2014). Additionally, discrimination and marginalization can affect how immigrants perceive and interact with healthcare services, potentially influencing their patterns of ED utilization. For instance, experiences of bias or exclusion in primary care settings may lead some immigrants to delay seeking care until an emergency arises, resulting in greater reliance on ED services (Krieger, 2014). Social exclusion and precarious living conditions further contribute to the exposure of immigrants to health risks, increasing their likelihood of needing ED care while also making them more vulnerable to receiving lower-quality treatment (Krieger, 2014).

Andersen’s model explores the conditions that influence individuals’ decisions to seek healthcare services. Healthcare utilization is shaped by predisposing factors, such as age, sex, and cultural attitudes, that affect the likelihood of seeking emergency care. It is also influenced by enabling factors such as health insurance coverage, income, and proximity to health services. In addition, need factors, such as the severity of a health condition, play a critical role. Different evaluation of these factors may lead to disparities between immigrants and Swiss citizens (Andersen, 1995).

By integrating these frameworks, this research explores how systemic inequalities (SDoH) interact with individual behaviors and resources (Andersen’s model) to influence disparities in access to healthcare. SDoH are used to contextualize the social and structural environment in which immigrants make healthcare decisions, while Andersen’s model allows us to examine how predisposing, enabling, and need factors shape these decisions. Together, these frameworks help identify not only whether disparities in ED use exist, but also the pathways through which they arise. This perspective is essential to consider the potential impact of social and structural factors on patterns of ED utilization among different population groups.

## 4 Data and methods

### 4.1 Data

Our study uses data from the most recent waves of the Swiss Health Survey (SHS), provided by the Federal Statistical Office (OFS).^3^ The SHS is a repeated cross-sectional survey that collects information on population’s health status and its determinants, the consequences of illnesses, the use of healthcare services, and insurance conditions. While the cross-sectional design does not allow for tracking individual trajectories over time, the regular data collection enables comparisons across survey waves and supports the evaluation of population-level changes and health policy measures. The survey has been conducted every five years since 1992 and collects data through telephone interviews and written questionnaires available in German, French, and Italian. Every wave is based on a representative random sample of households at the cantonal level and targets (different) individuals aged 15 and older living in private households across Switzerland. Alongside various sociodemographic characteristics, respondents are also asked to self-report their migration status. Our analysis focuses on the 2017 and 2022 waves as they provide the most recent data and include the complete set of variables relevant to our study.

### 4.2 Empirical approach

We used the SHS data to examine the differences in the use of ED between natives and immigrants. Our main outcome is self-reported ED attendances. This variable measures the number of ED attendances (including visits to hospitals, medical centers, or polyclinics^4^) within the 12 months preceding the survey. It has been recoded as 0 for no attendances (No) and 1 for one or more attendances (Yes). This recoding was necessary because the number of individuals who reported more than one ED attendances was very low, making it difficult to analyze the variable in its original count form.

Our focus is on differences across three population groups: first-generation immigrants, second-generation immigrants, and Swiss natives. First-generation immigrants are defined as individuals born outside of Switzerland to foreign-born parents. Second-generation immigrants are defined as individuals born in Switzerland to parents who were both born abroad.^5^ Migration status is self-reported in the latest SHS waves.

Firstly, we measure differences between Swiss and first- or second-generation immigrants controlling for a set of basic socioeconomic characteristics. Our baseline specification reads as follows:

(1) $$ED_{i}=\alpha +\beta \;{\text{ImmStatus}}_{i}+\gamma X_{i}+\psi \;{\text{Canton}}_{i}+\tau \;{\text{Year}}_{i}+\epsilon_{i}$$

where $ED_{i}$ is a binary variable equal to 1 if the individual reported having used the ED at least once during the 12 months prior to the survey date, and 0 otherwise. $ImmStatus_{i}$ is a categorical variable indicating the respondent’s immigrant status with three levels: 1) Swiss native (our reference level), 2) first-generation, 3) second-generation. $Canton_{i}$ and $Year_{i}$ are fixed effects for the respondent’s Canton of residence and the survey year. To isolate the role of immigration status, in the vector $X_{i}$ we control for a wide range of socioeconomic characteristics, including age cohort (15–24, 25–34, 35–44, 45–54, 55–64, 65–74, 75+), gender, highest level of education, marital status, employment, net household income, region type (urban, rural, peri-urban), number of kids under 17 years old in the household (details on the variables are provided in Table A1). $\epsilon_{i}$ is an idiosyncratic error term. Our coefficient of interest is represented by the vector β, which captures the differential propensity to visit the ED between Swiss natives and first- or second-generation immigrants.

Secondly, we explore factors that may explain differences in ED use between Swiss respondents and first- and second-generation immigrants. We do so by following a step-wise approach, adding separate additional sets of covariates capturing different individual characteristics that might explain differences between Swiss and immigrant respondents. Specifically, we run our main model specification in (1) by iteratively including different sets of control variables $M_{i}^{z}$ in addition to socioeconomic controls $X_{i}$. The groups of covariates $M_{i}^{z}$ were selected based on a combination of the conceptual frameworks guiding this study and the variables available in the SHS. The choice reflects factors identified in the SDoH and Andersen’s Model as potentially influencing ED utilization while also considering the practical constraints of data availability. We then explore the extent to which our main estimates for β change after including each set $M_{i}^{z}$ to gauge the extent to which it explains away part of the differences in propensity to use ED previously captured by immigrant status alone. Formally, we estimate the following extended specification

(2) $$ED_{i}=\alpha +\beta \;{\text{ImmStatus}}_{i}+\mu M_{i}^{z}+\gamma X_{i}+\psi \;{\text{Canton}}_{i}+\tau \;{\text{Year}}_{i}+\epsilon_{i}$$

We use the following three sets of additional control variables $M_{i}^{z}$ (details on the variables are provided in Table A2):

• Health status (z=1): self-reported health status (very bad, bad, fair, good, very good), body mass index (score), hypertension (yes/no), high cholesterol (yes/no).
• Health behaviors (z=2): smoking habits (non-smoker, occasional smoker, daily smoker), alcohol consumption (1 = more than twice a week, 0 = less than twice a week), and physical activity (inactive, partially trained (1–2 times per week), trained (3 or more times per week)).
• Healthcare use (z=3): number of visits to general practitioner/family doctor and specialist, number of asked pharmaceutical advice in the previous year.

If our sets of variables $M_{i}^{z}$ are predictors of ED use independent of immigration status, we expect β to shrink substantially and become statistically indistinguishable from zero. Whilst the proposed $M_{i}^{z}$’s are unlikely to be colliders or “bad controls” (Cinelli et al., 2024), the variables could potentially be observable confounders. In this case, we expect the estimated vector β to be similar to specification (1) and potentially to be estimated more precisely. In this latter case, under the assumption that unobserved confounding is proportional to confounding from observable sources (Oster, 2019), estimating specification (2) with different sets $M_{i}^{z}$’s and finding coefficient stability also serves as a loose robustness check against different potential sources of unobservable confounding.

Given the binary nature of our dependent variable, for both specifications we employ generalized linear model (GLM) with a binomial family and a logit link function estimated via maximum likelihood. Estimates are reported as average partial effects. All analyses were conducted using the statistical software R (packages *glm* and *margins*).

## 5 Results

### 5.1 Descriptive statistics

Table 1 describes individual characteristics of our complete study sample, which comprises a total of 16’183 observations spanning the years 2017 and 2022. Differences across different immigration statuses are tested using a joint F-test for numeric variables and a Chi-square ($\chi^{2}$) test for categorical variables. The results show significant differences across our groups of interest for various individual characteristics.

The sample is approximately equally split between men and women. Second-generation respondents were substantially younger: 32.1% were aged between 15 and 24, compared to 5.3% and 12.9% for first-generation and Swiss natives respectively. Only 2.3% of second-generation respondents were aged 75 or above. The main differences in education are related to higher education and professional or vocational training. 28.9% of first-generation individuals report having a higher education degree, compared to only about 17.2% and 17.6% for second-generation and Swiss natives. On the other hand, Swiss natives are substantially more likely to report vocational training (44.9%) compared to first-generation individuals (28.2%). In our sample, first- and second-generation immigrants are more likely to be employed, whilst Swiss natives are more likely to be inactive. First- and second-generation immigrants had a higher proportion of individuals reporting one or more ED visits in the 12 months prior to the survey response, with 18.5% and 21.7%, respectively, compared to 16.1% among Swiss natives.

**Table 1:** Descriptive statistics for core characteristics across migration status with pairwise tests.

Variable	*Swiss*			*First generation*			*Second generation*			Overall Test	Swiss vs First	Swiss vs Second
	N	Mean/%	SD	N	Mean/%	SD	N	Mean/%	SD			
ED attendances	10 987			4040			1156			X2 $=30.73^{***}$	X2 $=12.07^{**}$	X2 $=23.26^{***}$
... Yes	1770	16.1%		748	18.5%		251	21.7%				
... No	9217	83.9%		3292	81.5%		905	78.3%				
Gender	10 987			4040			1156			X2 $=2.71^{}$	X2 $=0^{}$	X2 $=2.545^{}$
... Man	4584	41.7%		1686	41.7%		511	44.2%				
... Woman	6403	58.3%		2354	58.3%		645	55.8%				
Age group	10 987			4040			1156			X2 $=1174.711^{***}$	X2 $=495.218^{***}$	X2 $=621.791^{***}$
... 15–24	1422	12.9%		214	5.3%		371	32.1%				
... 25–34	1110	10.1%		545	13.5%		199	17.2%				
... 35–44	1270	11.6%		815	20.2%		160	13.8%				
... 45–54	1799	16.4%		898	22.2%		257	22.2%				
... 55–64	1994	18.1%		681	16.9%		107	9.3%				
... 65–74	1955	17.8%		466	11.5%		35	3%				
... 75+	1437	13.1%		421	10.4%		27	2.3%				
Civil status	10 987			4040			1156			X2 $=513.439^{***}$	X2 $=139.399^{***}$	X2 $=298.58^{***}$
... Single	3198	29.1%		832	20.6%		611	52.9%				
... Divorced	1066	9.7%		408	10.1%		100	8.7%				
... Married	6015	54.7%		2601	64.4%		432	37.4%				
... Widowed	708	6.4%		199	4.9%		13	1.1%				
Education level	10 987			4040			1156			X2 $=668.714^{***}$	X2 $=643.554^{***}$	X2 $=53.991^{***}$
... Compulsory school	1613	14.7%		977	24.2%		243	21%				
... General education	984	9%		446	11%		140	12.1%				
... Tert: higher education	1939	17.6%		1167	28.9%		199	17.2%				
... Tert: professional training	1518	13.8%		311	7.7%		129	11.2%				
... Vocational training	4933	44.9%		1139	28.2%		445	38.5%				
Net income	10 987	3494	5832	4040	3397	5661	1156	3316	5780	F $=0.78^{}$	F $=0.819^{}$	F $=0.975^{}$
Employment status	10 987			4040			1156			X2 $=388.778^{***}$	X2 $=199.405^{***}$	X2 $=218.001^{***}$
... Apprentice	367	3.3%		59	1.5%		90	7.8%				
... Inactive	4131	37.6%		1330	32.9%		246	21.3%				
... Employee	5198	47.3%		2178	53.9%		688	59.5%				
... Entrepreneur	265	2.4%		82	2%		15	1.3%				
... Family business	285	2.6%		56	1.4%		22	1.9%				
... Independent	616	5.6%		193	4.8%		54	4.7%				
... Unemployed	125	1.1%		142	3.5%		41	3.5%				

Statistical significance markers: * p<0.1; ** p<0.05; *** p<0.01.

In Table 2, we report the differences between Swiss natives, first- and second-generation immigrants for our groups of predictors $M_{i}^{z}$. The mean values of numeric variables by immigration status reveal some notable differences. Most participants report being in good or very good health, with 37.7% of Swiss, 30.8% of first-generation, and 40.1% of second-generation immigrants, respectively. Very bad or bad health is relatively uncommon in all groups. First-generation immigrants tend to have a slightly higher BMI compared to both second-generation immigrants and Swiss nationals. Second-generation immigrants tend to have lower indicators of physical health, such as hypertension and high cholesterol. In relation to healthcare use, first-generation immigrants have more GP and specialist consultations on average. The frequency of pharmaceutical advice is somewhat higher among second-generation immigrants. Our sample also shows a mixed picture in terms of health behaviors across immigrant groups. Swiss natives demonstrate higher alcohol consumption than both first- and second-generation immigrants. Second-generation immigrants have a higher prevalence of daily smoking compared to both first-generation immigrants and Swiss natives, the latter of whom exhibit a higher rate of non-smoking. Furthermore, Swiss natives are more likely to engage in physical activity, while first-generation immigrants show a greater tendency toward physical inactivity.

**Table 2:** Differences in sets of additional predictors by migration status with pairwise tests.

Variable	*Swiss*			*First generation*			*Second generation*			Overall Test	Swiss vs First	Swiss vs Second
	N	Mean/%	SD	N	Mean/%	SD	N	Mean/%	SD			
Health status	10 987			4040			1156			X2 $=197.66^{***}$	X2 $=166.924^{***}$	X2 $=12.042^{**}$
... Very bad	41	0.37%		49	1.21%		4	0.35%				
... Bad	261	2.38%		179	4.43%		21	1.82%				
... Fair	1376	12.53%		701	17.35%		108	9.34%				
... Good	5164	47.0%		1866	46.19%		560	48.46%				
... Very good	4145	37.7%		1245	30.81%		463	40.05%				
BMI	10 987	24.7	4.47	4040	25.4	4.69	1156	24.5	4.48	F $=33.885^{***}$	F $=61.887^{***}$	F $=1.277^{}$
Hypertension	10 987			4040			1156			X2 $=98.432^{***}$	X2 $=8.319^{***}$	X2 $=94.983^{***}$
... No	8359	76.1%		3165	78.3%		1026	88.8%				
... Yes	2628	23.9%		875	21.7%		130	11.2%				
High cholesterol	10 987			4040			1156			X2 $=38.664^{***}$	X2 $=1.833^{}$	X2 $=33.238^{***}$
... No	8520	77.5%		3090	76.5%		982	84.9%				
... Yes	2467	22.5%		950	23.5%		174	15.1%				
Specialist consultations	10 987	1.81	4.74	4040	1.88	4.69	1156	1.81	5.42	F $=0.294^{}$	F $=0.588^{}$	F $=0.004^{}$
GP consultations	10 987	2.7	3.93	4040	3.02	4.34	1156	2.79	3.8	F $=9.129^{***}$	F $=18.106^{***}$	F $=0.49^{}$
Asked pharmaceutical advice	10 987	1.07	2.38	4040	1.14	2.92	1156	1.49	4.03	F $=12.921^{***}$	F $=2.277^{}$	F $=27.242^{***}$
Alcohol consumption	10 987			4040			1156			X2 $=191.01^{***}$	X2 $=108.765^{***}$	X2 $=114.625^{***}$
... No	4043	36.8%		1866	46.2%		612	52.9%				
... Yes	6944	63.2%		2174	53.8%		544	47.1%				
Smoking status	10 987			4040			1156			X2 $=96.17^{***}$	X2 $=59.913^{***}$	X2 $=49.858^{***}$
... Non-smoker	8538	77.7%		3008	74.5%		797	68.9%				
... Occasional smoker	819	7.5%		234	5.8%		102	8.8%				
... Daily smoker	1630	14.8%		798	19.8%		257	22.2%				
Physical activity	10 987			4040			1156			X2 $=158.608^{***}$	X2 $=151.232^{***}$	X2 $=2.668^{}$
... Inactive	710	6.5%		498	12.3%		73	6.3%				
... Partially trained	1781	16.2%		714	17.7%		209	18.1%				
... Trained	8496	77.3%		2828	70%		874	75.6%				

Statistical significance markers: * p<0.1; ** p<0.05; *** p<0.01.

### 5.2 Main results

In Table 3 we report our main findings, which indicate that immigration status is positively associated with ED attendances.

For first-generation immigrants, the association is statistically significant in the Unadjusted model (1) and remains significant in the Main model (2) as well as in the Health Behaviors model (4) and Healthcare Use model (5). The magnitude of the difference ranges from 0.016 to 0.020. The model where we account for Health Status (3) suggests no independent difference between Swiss natives and first-generation immigrants. For second-generation immigrants, the association is consistently positive. In the Unadjusted model (1), second-generation immigrants are 4.5% more likely to visit an ED (*p* < 0.001). The effect remains statistically significant in all the models, with coefficients ranging from 2.4% to 3.0% (*p* < 0.05). However, when controlling for Healthcare Use (5), the difference decreases slightly to 2.2% and becomes statistically not significant.

**Table 3:** Coefficient estimates of the effect of immigration status on ED use.

	(1)	(2)	(3)	(4)	(5)
	Unadjusted	Main	Health Status	Health Behaviors	Healthcare Use
*Immigration status*					
First generation (vs. Swiss natives)	0.025***	0.020**	0.007	0.016*	0.016*
	(0.004)	(0.006)	(0.006)	(0.006)	(0.007)
Second generation (vs. Swiss natives)	0.045***	0.030**	0.024*	0.027**	0.022
	(0.007)	(0.010)	(0.010)	(0.010)	(0.011)

*Predictor sets*					
Health status					
...Bad			−0.119*		
			(0.053)		
...Fair			−0.268***		
			(0.050)		
...Good			−0.352***		
			(0.049)		
...Very good			−0.398***		
			(0.049)		
Body mass index			0.001		
			(0.001)		
High cholesterol			0.006		
			(0.007)		
Hypertension			0.025**		
			(0.008)		
Physical activity					
...Partially trained				−0.059***	
				(0.012)	
...Trained				−0.055***	
				(0.011)	
Alcohol consumption				−0.019***	
				(0.005)	
Smoking habits					
...Occasional smoker				0.005	
				(0.010)	
...Daily smoker				0.026***	
				(0.007)	
Asked pharmaceutical advice					0.005***
					(0.001)
GP consultation in previous year					0.012***
					(0.001)
Specialist consultation in previous year					0.005***
					(0.001)

Socioeconomic controls	NO	YES	YES	YES	YES

McFadden’s R${}^{2}$	0.002	0.015	0.043	0.019	0.048
Log Likelihood	−18249.777	−8524.967	−8039.107	−8374.683	−7337.017
AIC	36 505.554	17 153.934	16 196.215	16 863.365	14 784.034
BIC	36 531.508	17 566.445	16 662.234	17 314.643	15 209.444

Observations	42 238	20 597	19 903	20 275	16 894

Note: All models estimated using a GLM estimator with logit link function. Coefficients represent average partial effects. Robust standard errors in parentheses. Stars indicate statistical significance as follows: ∗∗∗p<0.001; ∗∗p<0.01; ∗p<0.05.

### 5.3 Heterogeneity analysis

We ran additional analyses to explore potential dimensions of heterogeneity in our results.

Firstly, immigrants are not a homogeneous group, as their cultural norms, healthcare-seeking behaviors and socioeconomic conditions can vary significantly depending on their regions of origin. These factors directly influence their health needs and utilization of healthcare services, including ED visits. For example, migrants from certain regions may experience specific health conditions or face distinct challenges in accessing healthcare, which shape their patterns of ED use (Malmusi et al., 2010). We substituted our main exposure $ImmStatus_{i}$ with an alternative variable, ${\mathrm{Origin}}_{i}$ as provided in the SHS, which categorizes migrants into the five world regions of origin (Table 4).

We ran regression models to examine how the region of origin influences ED attendances, using the same variables as those included in the regression models but substituting for migration status (Table A3). The corresponding estimating equation reads as follows:

**Table 4:** Main regions of origin of immigrants in Switzerland.

Region of origin	N	%
Switzerland (CH)	13 230	81.75
North-West Europe (NW)	862	5.33
South-East and East Europe (SEE)	694	4.29
South-West Europe (SW)	1186	7.33
Rest of the world (RoW)	211	1.30

Total	16 183	100.00

(3) $$ED_{i}=\alpha +\beta \;{\text{Origin}}_{i}+\mu M_{i}^{z}+\gamma X_{i}+\psi \;{\text{Canton}}_{i}+\tau \;{\text{Year}}_{i}+\epsilon_{i}$$

In the Unadjusted model (1) comparing ED attendances to regions of origin, positive and significant associations are observed for all categories except for NW Europe, which shows a negative association that aligns with the socio-economic and healthcare similarities between NW Europe and Switzerland. Immigrants from SEE and SW Europe have significantly higher probabilities of ED visits (*p* < 0.001), while those from RoW show a smaller but still significant increase (*p* < 0.05). Immigrants from RoW are 3.1%, those from SEE are 6.4%, and those from SW are 3.6% more likely to visit ED.

In the “Main” model (2), significant associations remain only for SEE and SW Europe immigrants (*p* < 0.05). Specifically, SEE immigrants are 4.6% more likely to visit the ED, while SW immigrants are 3.7% more likely. In Column 3, SEE immigrants show a 3.1% higher likelihood of ED visits compared with Swiss natives. In Column 4, the estimates rise to 3.8% for SEE immigrants and 3.0% for those from SW Europe. Column 5 reports similar patterns, with SEE and SW Europe immigrants showing 3.7% and 3.6% higher likelihoods, respectively.

We then examined the interaction between gender and immigration status, exploring whether the relationship between immigration status, and the outcome variable, ED attendances, depends on the gender of the individual (Table A4). Our empirical specification for this heterogeneity analysis reads as follows:

(4) $$ED_{i}=\alpha +\beta \;{\text{ImmStatus}}_{i}+\phi \;{\text{Sex}}_{i}+\xi \left({\text{ImmStatus}}_{i}\times {\text{Sex}}_{i}\right)\;+\;\gamma X_{i}+\psi \;{\text{Canton}}_{i}+\tau \;{\text{Year}}_{i}+\epsilon_{i}$$

The results suggest that an interaction exists between sex and immigration status in relation to ED attendances. In the Unadjusted model (1), first- and second-generation immigrant women report 2.6% and 6.8% higher ED use, respectively, compared to Swiss male natives (*p* < 0.001). For first-generation immigrant women, the association remains significant only in the Main (2) and Health behaviors (4) models and is not significant elsewhere. In contrast, second-generation immigrant women consistently exhibit higher ED use than Swiss male natives across all model specifications.

### 5.4 Robustness checks

To support the robustness of our findings, we propose a battery of robustness and sensitivity checks.

Firstly, we decompose the difference between Swiss, first- and second-generation migrants using a non-linear multivariate Oaxaca-Blinder decomposition. We follow the approach in Tzogiou et al. (2021) and unpick the explained and unexplained parts of the difference in ED use between Swiss natives and the two immigrant groups, reporting percentage contributions of each group of variables. Albeit less flexible than our regression approach, this analysis takes a more explicit approach to unpicking the role of different variables in determining the contribution to observed average differences in ED use between our groups of interest. To implement the analysis, we use the same variables outlined in our main approach, and conduct a “twofold” decomposition in a component explained by differences in endowments (e.g. health), and in an unexplained part of the differences (related to differential responses or unobserved factors). We employ the R package *oaxaca* with a GLM estimator and a logit link function (Hlavac, 2022). The results of this approach are reported in Figure A1 and Table A5. The findings are fully aligned with our main analysis and suggest that differences in ED use between Swiss and first-generation immigrants are mainly explained by differences in health status. On the other hand, differences between Swiss and second-generation immigrants are mostly unexplained by our variables, with some influence for pharmacy advice. For both immigrant groups, compared to Swiss natives, the age structure is a major determinant of differences in ED use.

Secondly, our analysis overlooks the role of health insurance coverage, which may well be correlated with immigration status. To ensure the extent to which this may drive our results, we ran an analysis analogous to our main specification in (2) but focused only on the data from 2017 to study insurance-related information. This approach was necessary because the 2022 dataset does not include information on insurance type, deductible and the hospital ward insured, which were available in the 2017 dataset. By isolating the 2017 data, we ensured that all the variables related to insurance type were included in the analysis, allowing us to explore how differences in insurance coverage influenced ED visits specifically for that year.

The initial column (Table A6) considers only ED attendances and migration status in 2017, revealing that first- and second-generation immigrants experience positive and highly significant differences of 2.9% and 5.1% (1), respectively, compared to Swiss natives (*p* < 0.001). In Columns 2 to 4, the difference remains significant only for second-generation immigrants (*p* < 0.05), showing that they are 2.6%, 2.5%, and 2.4% more likely to visit the ED compared to Swiss natives. Meanwhile, in the “Insurance Type” model (5), which includes variables such as the insurance model, annual deductible amount, and type of hospital ward covered, the differences for both first- and second-generation immigrants are sensibly reduced close to zero and not significant. Similarly, we find no significant differences in the “Healthcare Use” column (6).

Third, we assessed the robustness of our results by estimating all models from the main analysis on a sample that is consistent across the step-wise inclusion of our three sets of predictors (16’183 observations), ensuring full comparability of estimates. The results (Table A7) confirm the stability of our estimates, as the main findings remain almost unchanged. Second-generation immigrants remain more likely to visit the ED, with the differential increasing from 4.5% to 5.6% (*p* < 0.001).

Additionally, we use this consistent sample of 16’183 observations to estimate a model where we include all sets of control and predictors variables simultaneously (Table A8). This approach ensures that the findings are consistent with different assumptions. In the Unadjusted model (1), the coefficients for both first- and second-generation immigrants are significant (*p* < 0.001). In the subsequent models, only the coefficient for second-generation immigrants remains significant (*p* < 0.05), although it becomes insignificant in Model 5. These results confirm the stability of our estimates, as the main findings remain almost unchanged.

Finally, we performed two additional checks assuming a linear probability model and an ordinary least square (OLS) estimator as an alternative to a generalized linear model (GLM). We first compare the results from these two different estimation approaches on our main empirical specification. The outcomes from both models were similar and consistent (Table A9). As in the GLM model, the coefficient for first-generation immigrants is statistically significant only in the Unadjusted model. For second-generation immigrants, the coefficients remain almost the same in both the GLM and LM models.

## 6 Discussion

This study examined disparities in Emergency Department utilization between Swiss natives, first- and second-generation immigrants, and explored the factors most likely explaining these differences. Our findings challenge the common assumption that immigrants, particularly first-generation immigrants, disproportionately rely on ED services due to barriers such as language difficulties or limited knowledge of the healthcare system Norredam et al., 2004, Hjern et al., 2001, Rué et al., 2008.

Our first observation concerns first-generation immigrants. We find that the difference in ED use between first-generation immigrants and Swiss natives disappears when accounting for differences in health status. Interestingly, in our sample, first-generation immigrants are less healthy compared to non-immigrants. On the other hand, second-generation immigrants show a more nuanced pattern: differences with Swiss natives persist even after controlling for sociodemographic characteristics, health status, and health behaviors. Only when controlling for proxies of use of other healthcare services we measure a drop in the difference from 2.7 to 2.2 percentage points, a relative drop of about 18 percent.

These findings about first-generation immigrants challenge the *Healthy Immigrant Effect* theory, mainly because our first-generation immigrants are less healthy than Swiss natives. Under the *Healthy Immigrant Effect* hypothesis, several factors could have explained this phenomenon. Immigrants tend to be younger, predominantly male individuals. ED utilization generally declines with age, and younger individuals are more prone to engage in risk-taking behaviors (Hernández-Quevedo and Jiménez-Rubio, 2009). Moreover, in Switzerland, nearly half of the working-age immigrant population is highly educated (Hercog and Cangià, 2021). Higher educational attainment among immigrants is linked to better health outcomes and a lower likelihood of ED use. Greater education also typically translates into better awareness of healthcare options, preventive care and overall health management (Wittink and Oosterhaven, 2018). While these factors illustrate why first-generation immigrants may not experience differences in ED utilization, other elements can come into play over time and filter through to the second generation. Immigrants who integrate into the host society (e.g., mastering the local language or living in Switzerland for an extended period) tend to adopt less healthy behaviors, thereby potentially increasing their risk of health complications over time (Bodenmann et al., 2010). This gradual change aligns with the concept of health assimilation, or the *exhausted immigrant effect*, where the initial health advantage of immigrants diminishes as they adapt to their new environment (Ayala-Diaz et al., 2025, Giuntella and Mazzonna, 2015).

In fact, a different pattern emerges when examining second-generation immigrants (Table 3). While all models yield statistically significant results (*p* < 0.05), the “Healthcare Use” model (5) stands out as the exception. This model shows that, for the reference group (Swiss natives), GP visits, specialist consultations and requests for pharmaceutical advice are significantly associated with ED utilization. However, the coefficient for second-generation immigrants in this model falls just short of conventional statistical significance (*p* < 0.05). Results from an alternative approach to decompose the difference in ED use between Swiss natives and second-generation migrants are broadly consistent with these findings.

Although research on ED utilization among second-generation immigrants yields mixed results, our findings suggest that individuals with higher overall healthcare use are also more likely to visit EDs than Swiss natives. On the one hand, some studies suggest that a higher degree of acculturation among second-generation immigrants leads to healthcare utilization patterns similar to or even lower than those of native populations (Kao, 2009, Steinhausen et al., 2009, Burgos et al., 2005, Rumbaut, 2004, Leclere et al., 1994), in contrast to our findings. Other studies emphasize the role of structural and socioeconomic disadvantages, such as poorer health status, lower socioeconomic position, and limited social support, as key factors that may drive greater use of both primary and secondary care among second-generation immigrants (Volken and Rüesch, 2014).

In the Swiss context, research has shown that second-generation immigrants experience greater well-being when they are both oriented toward Swiss culture and maintain ties to their heritage culture. These bicultural dynamics may influence not only subjective well-being but also healthcare utilization patterns, potentially explaining the higher ED use observed among some second-generation individuals in our sample (Schwarz and Pfammatter, 2024). Specifically, those who successfully navigate both cultural frameworks may feel more confident in interacting with healthcare providers and interpreting health information, while others may experience cultural tension or uncertainty when seeking care. Eytan et al., 2007, Mantwill and Schulz, 2017.

The findings from the “Regions of origin” models align with existing evidence showing that immigrants from SEE and SW Europe tend to use ED services more frequently than native populations. In several European contexts, such as Spain, EDs have been described as a key entry point into the healthcare system for immigrant groups with limited access to specialized or primary care (Hernández-Quevedo and Jiménez-Rubio, 2009). Similarly, in Switzerland, SEE and SW European immigrants are more likely to rely on walk-in ED services compared to Swiss patients, suggesting that EDs may compensate for structural or informational barriers in navigating the healthcare system (Klukowska-Röetzler et al., 2018). Conversely, immigrants from NW Europe display healthcare utilization patterns comparable to those of Swiss nationals, indicating that proximity in healthcare systems and cultural familiarity may facilitate more equal access to services (Lay et al., 2006). Finally, the higher rates of ED utilization among non-European immigrants observed in other countries (Sandvik et al., 2012) are consistent with our findings, reinforcing the notion that differences in access pathways and systemic barriers, rather than medical need alone, contribute to cross-group disparities in ED use.

When modeling for 2017 (Table A1), the “Insurance Type” (5) model shows that the coefficients for immigration status are not significant, though the negative coefficient for first-generation immigrants is noteworthy. Insurance coverage plays a crucial role in healthcare disparities, particularly among immigrants from lower socio-economic backgrounds, may hinder timely care, potentially increasing ED reliance for urgent needs (Tzogiou et al., 2021). These findings highlight the dual role of financial constraints: while high deductibles reduce ED visits by discouraging unnecessary care, inadequate insurance coverage can limit access to primary care, leading to greater dependence on ED services.

To better understand how immigration status relates to ED use, we introduced an interaction between gender and immigration status (Table A3). The results show that second-generation immigrant women consistently report higher ED use compared to Swiss native men across all models (*p* < 0.01). In contrast, the interaction term for first-generation immigrant women becomes insignificant once controls are added. These findings indicate that the higher ED use among second-generation immigrants is primarily driven by women. Gender differences in healthcare utilization show that women tend to use ED services more often, partly due to higher levels of mental distress and a greater willingness to seek help, whereas men often underutilize services due to societal expectations around masculinity (Koopmans and Lamers, 2007). This supports previous evidence suggesting that gender-specific roles, cultural expectations, and migration-related challenges intersect to influence how men and women access and use emergency care services (Gerritsen and Devillé, 2009). While our analysis focuses on Switzerland, a recent nationwide study in China found that stronger social integration was positively associated with primary healthcare utilization among internal migrants, suggesting that higher integration may shift care-seeking behavior away from emergency care and toward more appropriate primary care services (Wang et al., 2023).

This study contributes to the existing literature on healthcare utilization disparities by offering new insights into the Emergency Department usage patterns of Swiss natives and immigrants. While much of the previous research has focused on the higher reliance of first-generation immigrants on ED services, our findings reveal a more subtle picture that goes beyond the current state of research. This complexity adds layers to the potential social implications and policy applications.

With our results, we challenge the general assumption that first-generation immigrants disproportionately utilize ED services due to barriers such as language, socio-economic disadvantages, and lack of healthcare system knowledge (Hernández-Quevedo and Jiménez-Rubio, 2009, Klukowska-Röetzler et al., 2018). Instead, our study highlights that a broader set of factors may play more significant roles in shaping healthcare utilization patterns. For second-generation immigrants, our results contrast with previous studies that suggest their healthcare utilization patterns may mirror those of the native population as they become more integrated in the host country society (Kao, 2009, Steinhausen et al., 2009). While integration may lead to similar healthcare behavior, we also underscore the importance of socioeconomic determinants and health status in influencing ED visits, suggesting that disparities may persist even among those who are more integrated into the host society. Our results are consistent with the findings of Tzogiou et al. (2021) (Tzogiou et al., 2021). However, our study goes one step further by examining a broader range of factors beyond immigrant status alone, including intersection with gender, allowing for a more detailed understanding of the determinants of ED utilization.

Our study has some limitations. First, the Swiss Health Survey (SHS) relies on self-reported data, which may be subject to recall bias or social desirability bias, potentially affecting the accuracy of healthcare utilization measures. Additionally, SHS is not ideally suited for examining differences in healthcare utilization between immigrants and non-migrants due to its language limitations. However, the broad national coverage of SHS allows for the inclusion of diverse individuals, which enhances the generalizability of the findings despite the language constraints. The sample is representative at the cantonal level, strengthening the reliability of the results. We also had intended to include religion as an additional variable to enhance the analysis, providing a cultural perspective that could help explain differences in healthcare service utilization; however, this information was last collected in the 2007 wave. The absence of religion in more recent data has not hindered our analysis significantly, as other relevant sociodemographic variables remain strong predictors of healthcare utilization. A further limitation of the SHS is its cross-sectional nature, meaning it does not allow for longitudinal analysis of healthcare utilization trends. However, this approach still provides valuable snapshots of healthcare access across diverse population groups, allowing for a comparison between immigrants and non-immigrants at a single point in time.

## 7 Conclusion

This study examined disparities in Emergency Department use between Swiss natives and immigrant populations. Our findings show that while first-generation immigrants use ED services at rates similar to natives, second-generation immigrants are more likely to use ED care. This suggests that the systemic barriers affecting immigrants may not lead to more ED use among first-generation individuals but become more relevant as immigrants settle and integrate over time.

Given that second-generation immigrants are increasingly likely to remain in Switzerland, understanding the patterns of healthcare utilization in this group remains important. While our study does not assess the appropriateness of emergency department use, identifying the factors associated with ED visits can help inform interventions to support equitable access and navigation of the healthcare system. Future research should seek to understand patterns of ED utilization from the perspective of migrants, capturing their experiences, perceptions, and potential barriers. In addition, studies should consider supply-side factors, such as provider practices, resource allocation, and organizational policies, to identify structural determinants of disparities.

In conclusion, policy interventions should be designed with the complexities highlighted in our study in mind. Efforts should focus on reducing access barriers and enhancing the affordability and continuity of care for immigrant populations. This can include expanding primary care outreach and preventive services, offering language support and cultural mediation, and increasing awareness about healthcare rights and available services. Tailored interventions, particularly in areas with high concentrations of immigrants, may help reduce reliance on emergency department services and improve health outcomes. Special attention should be given to second-generation immigrants, who appear more vulnerable to the cumulative effects of socioeconomic and systemic disadvantages (Lebano et al., 2020, Szczepura, 2005).

## CRediT authorship contribution statement

**Ludovica Alesci:** Writing – original draft, Visualization, Validation, Software, Methodology, Investigation, Formal analysis, Data curation, Conceptualization. **Igor Francetic:** Writing – review & editing, Validation, Supervision, Project administration, Methodology, Funding acquisition, Conceptualization.

## Funding

This research was funded by the 10.13039/501100001711Swiss National Science Foundation (SNSF), Switzerland (grant number 216326).

## Declaration of competing interest

The authors declare that they have no known competing financial interests or personal relationships that could have appeared to influence the work reported in this paper.

## Appendix A Supplementary data

MMC S1 Tables and figures related to additional analyses. MMC S1

## Footnotes

1 Swiss Federal Statistical Office (OFS). Population by migration status. 16.12.2024 https://www.bfs.admin.ch/bfs/en/home/statistics/population/migration-integration/by-migration-status.html.

2 Switzerland is organized as a federal state where power is divided among three administrative levels. The Confederation (national government), Cantons, and Municipalities. Switzerland is composed of 26 Cantons, each with its own government. Amongst other devolved powers, Cantons are responsible for the organization of the healthcare and education systems for their populations.

3 Swiss Federal Statistical Office (OFS). Enquête Suisse sur la santé. https://www.bfs.admin.ch/bfs/fr/home/statistiques/sante/enquetes/sgb.html.

4 In Switzerland, EDs are primarily located in hospitals and provide acute care for urgent medical conditions. Some medical centers and polyclinics also offer urgent outpatient care. Note that the SHS survey question does not distinguish between these different types of ED services.

5 It is important to note that this classification is one of many possible interpretations of immigrant status, as the definition of “immigrant” can vary depending on legal, social, or generational criteria.
