# Supplementary material for: Emergency Department utilization in Switzerland: Comparing Swiss natives with first- and second-generation immigrants
Source: J Migr Health. 2026 Jan 2;13:100388. doi: 10.1016/j.jmh.2025.100388 (PMC12809692; doi:10.1016/j.jmh.2025.100388)
Supplement: MMC S1 — Tables and figures related to additional analyses. [file mmc1.pdf]

Appendix to "Emergency Department utilization in Switzerland: comparing Swiss natives with First- and Second-Generation Immigrants"

Table A1: Control variables of the SHS questionnaire

**Variable**	**Categories / Codes**	**Question**
Regions of origin	1: Switzerland<br>2: Northern and Western Europe<br>3: Southwestern Europe<br>4: Eastern and Southeastern Europe<br>5: Outside Europe	Country of birth
Immigration status	1: Population without migration background<br>2: 1st generation<br>3: 2nd generation or more	If Country of birth = Switzerland and Nationality $\neq$ Switzerland $\rightarrow$<br>Was your mother born in Switzerland or abroad?<br>Was your father born in Switzerland or abroad?
Education level	1: Compulsory school<br>2: Upper secondary – vocational<br>3: Upper secondary – general<br>4: Tertiary – higher vocational<br>5: Tertiary – higher education	What is the highest level of education you have completed?
Civil status	1: Single<br>2: Married<br>3: Divorced<br>4: Widowed	Civil status
Age (7 groups)	1: 15–24<br>2: 25–34<br>3: 35–44<br>4: 45–54<br>5: 55–64<br>6: 65–74<br>7: 75+	Date of birth
Canton of residence	1: Zürich ... 26: Jura	ZIP code / Municipality
Gender	1: Men<br>2: Women	Who are you?
Net household monthly income	Numeric	Considering all sources of income, what is the total monthly net income (after social deductions) of your entire household?
ED attendances	Number	How many times in the last 12 months were you admitted to an emergency department?
Professional status	1: Self-employed<br>2: Employee of own SA/SAGL<br>3: Family business worker<br>4: Employee in private/public company<br>5: Apprentice	These questions refer to your main job. If you have more than one, answer for the most important one.
Number of children (17 years)	Number	Do you have children who no longer live in your household?
Urban/rural region	1: Urban<br>2: Intermediate (dense peri-urban and rural centers)<br>3: Rural	ZIP code / Municipality
Housing situation	1: Tenant – apartment/house/room<br>2: Tenant – cooperative housing<br>3: Owner/co-owner – house<br>4: Owner/co-owner – apartment<br>5: Other situation	Regarding your usual dwelling: do you or another person in your household own or rent it?

Table A2: Predictors of the SHS questionnaire

**Variable**	**Categories / Codes**	**Question**
Self-reported health status	1: Very bad<br>2: Bad<br>3: Fair<br>4: Good<br>5: Very good	How is your general health?
Body Mass Index	Score	Please indicate your BMI (Body Mass Index) score.
Hypertension	Yes/No	Has a doctor or someone working in the medical field ever told you that your blood pressure was too high?
High cholesterol	Yes/No	Has a doctor or other health professional ever told you that your cholesterol level is too high?
Smoking habits	1: Non-smoker<br>2: Occasional smoker<br>3: Daily smoker	How many cigarettes do you smoke per day on average?
Alcohol consumption	1: 3+ times/day<br>2: 2 times/day<br>3: 1 time/day<br>4: Several times/week<br>5: 1–2 times/week<br>6: 1–3 times/month<br>7: Less than once/month<br>8: Never, teetotal	How often do you usually consume alcoholic beverages (beer, wine, spirits, etc.)?
Physical activity	1: Inactive<br>2: Partially active (1–2 times per week)<br>3: Active (3+ times per week)	How often do you do gymnastics, fitness, or sports?
GP visits	Number	In the last 12 months, how many times have you visited a general practitioner?
Specialist visits	Number	In the last 12 months, how many times have you visited a specialist?
Asked pharmaceutical advice	Number	How many times in the last 12 months did you ask for advice in a pharmacy for a health problem?

Table A3: Coefficient estimates of the effect of the region of origin on ED use

	(1) Unadjusted	(2) Main	(3) Health Status	(4) Health Behaviors	(5) Healthcare Use
Region of origin					
North-West Europe	-0.001	-0.004	-0.006	-0.005	-0.014
	(0.007)	(0.011)	(0.011)	(0.011)	(0.013)
South-East and East Europe	0.064***	0.046***	0.031*	0.038**	0.037*
	(0.009)	(0.013)	(0.013)	(0.013)	(0.015)
South-West Europe	0.036***	0.037***	0.017	0.030**	0.036**
	(0.007)	(0.011)	(0.010)	(0.011)	(0.012)
Rest of the world	0.031*	0.035	0.030	0.035	0.038
	(0.015)	(0.022)	(0.022)	(0.022)	(0.026)
Predictor sets					
Health status					
...Bad			-0.116*		
			(0.054)		
...Fair			-0.264***		
			(0.050)		
...Good			-0.348***		
			(0.050)		
...Very good			-0.394***		
			(0.050)		
Body mass index			0.001		
			(0.001)		
High cholesterol			0.007		
			(0.007)		
Hypertension			0.024**		
			(0.008)		
Physical activity					
...Partially trained				-0.059***	
				(0.012)	
...Trained				-0.053***	
				(0.011)	
Alcohol consumption				-0.018***	
				(0.005)	
Smoking habits					
...Occasional smoker				0.005	
				(0.010)	
...Daily smoker				0.026***	
				(0.007)	
Pharmaceutical advice					0.005***
					(0.001)
GP consultation					0.012***
					(0.001)
Specialist consultation					0.005***
					(0.001)
Socioeconomic controls	NO	YES	YES	YES	YES
McFadden's R^2	0.002	0.015	0.043	0.019	0.048
Log Likelihood	-18351.721	-8546.574	-8060.929	-8397.263	-7355.219
AIC	36713.443	17201.147	16243.859	16912.526	14824.438
BIC	36756.722	17629.655	16725.825	17379.780	15265.458
Observations	42439	20647	19952	20324	16936

Note: All models estimated using a GLM estimator with logit link function. Coefficients represent average partial effects. Robust standard errors in parentheses. Stars indicate statistical significance as follows: $^{***}p < 0.001$; $^{**}p < 0.01$; $^{*}p < 0.05$.

Table A4: Coefficient estimates of the effect of immigration status on ED use with Gender-immigration interaction

	(1) **Unadjusted**	(2) **Main**	(3) **Health Status**	(4) **Health Behaviors**	(5) **Healthcare Use**
Immigration status					
...First generation	0.010	0.013	0.002	0.009	0.013
	(0.006)	(0.009)	(0.009)	(0.009)	(0.011)
...Second generation	0.016	0.006	-0.001	0.004	-0.009
	(0.011)	(0.014)	(0.014)	(0.014)	(0.016)
...First generation*women	0.026***	0.025**	0.011	0.021*	0.018
	(0.006)	(0.009)	(0.008)	(0.008)	(0.009)
...Second generation*women	0.068***	0.051***	0.047**	0.048***	0.046**
	(0.012)	(0.014)	(0.014)	(0.014)	(0.015)
Socioeconomic controls	NO	YES	YES	YES	YES
Predictor sets	NO	YES	YES	YES	YES
McFadden's R^2	0.002	0.015	0.044	0.019	0.048
Log Likelihood	-15998.873	-8522.446	-8036.382	-8372.198	-7333.934
AIC	32009.746	17152.893	16194.764	16862.396	14781.869
BIC	32060.988	17581.269	16676.581	17329.507	15222.748
Observations	37805	20597	19903	20275	16894

Note: All models estimated using a GLM estimator with logit link function. Coefficients represent average partial effects. Robust standard errors in parentheses. Stars indicate statistical significance as follows: $^{***}p < 0.001$; $^{**}p < 0.01$; $^{*}p < 0.05$.

	Δ First generation	Perc. explained	Δ Second generation	Perc. explained
Sociodemographics	-0.0006	-4.8508	0.0259	94.27
Health status	0.0078	59.79	-0.0033	-12.05
Health behaviors	0.0017	13.11	0.0015	5.67
Healthcare use	0.0042	31.95	0.0033	12.11
Total explained	0.0131	100.00	0.0275	100.00
Total unexplained	0.0109		0.0284	
Total difference	0.0241		0.0056	

Table A5: Percentage contribution of different sets of predictors from a twofold Oaxaca-Blinder decomposition

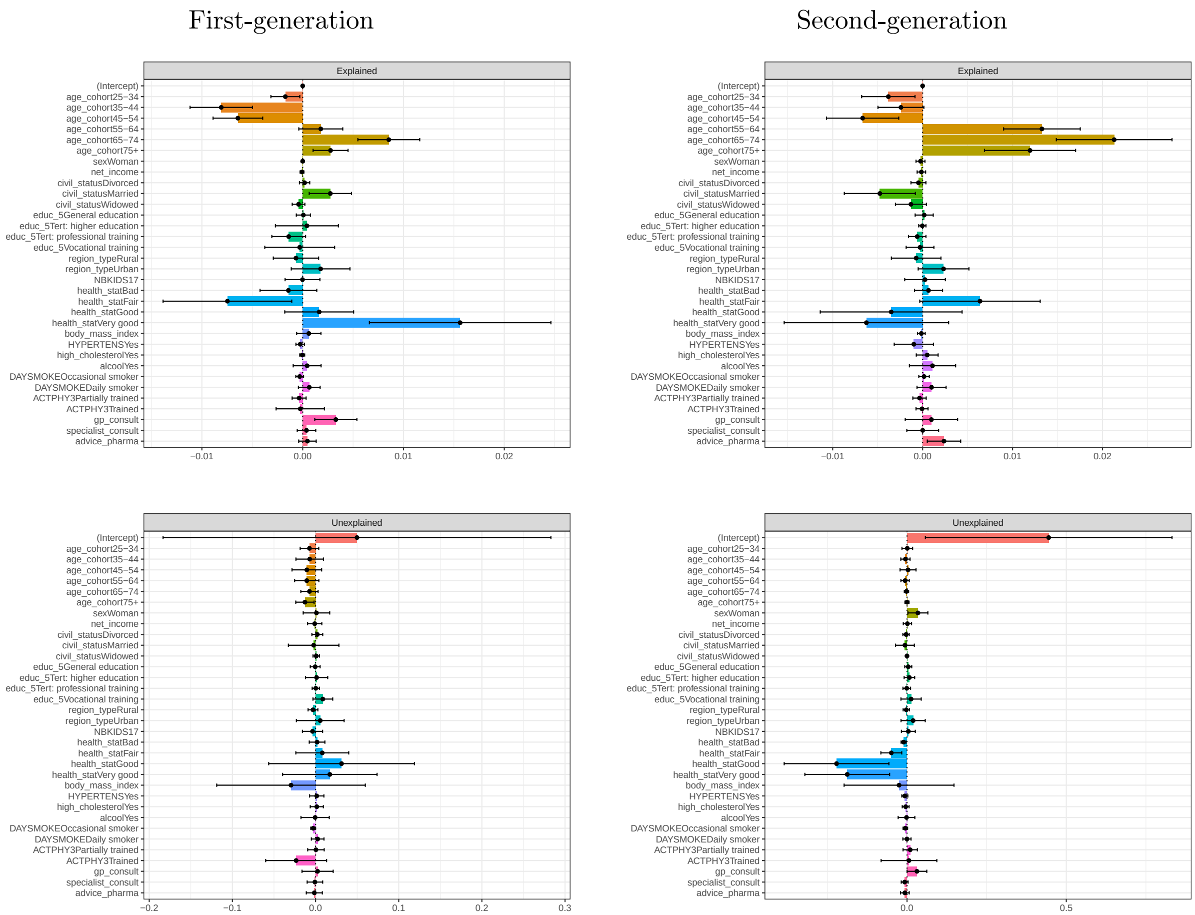

Figure A1: Results from Oaxaca-Blinder decomposition

Table A6: Coefficient estimates of the effect of immigration status on ED use 2017

	(1) Unadjusted	(2) Main	(3) Health Status	(4) Health Behaviors	(5) Insurance type	(6) Healthcare Use
Immigration status						
First generation	0.029***	0.016*	0.003	0.012	0.006	0.013
	(0.006)	(0.007)	(0.007)	(0.007)	(0.008)	(0.008)
Second generation	0.051***	0.029**	0.025*	0.025*	0.019	0.023
	(0.010)	(0.011)	(0.011)	(0.011)	(0.013)	(0.013)
Predictor sets						
Body mass index			0.001			
			(0.001)			
Health status						
...Bad			-0.086			
			(0.058)			
...Fair			-0.227***			
			(0.054)			
...Good			-0.319***			
			(0.054)			
...Very good			-0.361***			
			(0.054)			
High cholesterol			0.008			
			(0.008)			
Hypertension			0.030***			
			(0.009)			
Physical activity						
...Partially trained				-0.061***		
				(0.013)		
...Trained				-0.058***		
				(0.012)		
Alcohol consumption				-0.022***		
				(0.006)		
Smoking habits						
...Occasional smoker				0.001		
				(0.010)		
...Daily smoker				0.027***		
				(0.008)		
Deductible						
...500 CHF					-0.019*	
					(0.010)	
...1000 CHF					-0.033*	
					(0.014)	
...1500 CHF					-0.049***	
					(0.010)	
...2000 CHF					-0.040**	
					(0.014)	
...2500 CHF					-0.055***	
					(0.008)	
Hospital ward insured						
...General ward					-0.006	
					(0.011)	
...Private ward					-0.013	
					(0.012)	
Insurance type						
...Bonus Insurance					0.044	
					(0.043)	
...Family doctor model					-0.013*	
					(0.007)	
...HMO insurance					-0.034*	
					(0.015)	
...Pre-consultation telephone model					0.008	
					(0.011)	
Pharmaceutical advice						0.005***
						(0.002)
GP consultation in previous year						0.011***
						(0.001)
Specialist consultation in previous year						0.005***
						(0.001)
Socioeconomic controls	NO	YES	YES	YES	YES	YES
McFadden's R^2	0.002	0.015	0.044	0.020	0.049	0.022
Log Likelihood	-9025.400	-7045.538	-6649.498	-6946.354	-5560.320	-6034.660
AIC	18056.800	14195.077	13416.996	14006.708	11246.640	12179.319
BIC	18080.679	14597.961	13872.105	14447.553	11721.778	12594.225
Observations	21150	17116	16543	16884	13931	13957

Note: All models estimated using a GLM estimator with logit link function. Coefficients represent average partial effects. Robust standard errors in parentheses. Stars indicate statistical significance as follows: $^{***}p < 0.001$; $^{**}p < 0.01$; $^{*}p < 0.05$.

Table A7: Coefficient estimates of the effect of immigration status on ED use for a complete sample

	(1) **Unadjusted**	(2) **Main**	(3) **Health Status**	(4) **Health Behaviors**	(5) **Healthcare Use**
Immigration status					
...First generation	0.024***	0.022**	0.008	0.016*	0.017*
	(0.007)	(0.008)	(0.007)	(0.008)	(0.007)
...Second generation	0.056***	0.029*	0.025*	0.025*	0.023*
	(0.013)	(0.012)	(0.012)	(0.012)	(0.012)
Predictor sets					
Health status					
...Bad			-0.123*		
			(0.055)		
...Fair			-0.271***		
			(0.051)		
...Good			-0.350***		
			(0.051)		
...Very good			-0.393***		
			(0.051)		
Body mass index			0.001		
			(0.001)		
High cholesterol			0.001		
			(0.008)		
Hypertension			0.018*		
			(0.009)		
Physical activity					
...Partially trained				-0.059***	
				(0.014)	
...Trained				-0.053***	
				(0.012)	
Alcohol consumption				-0.023***	
				(0.006)	
Smoking habits					
...Occasional smoker				0.005	
				(0.012)	
...Daily smoker				0.030***	
				(0.008)	
Pharmaceutical advice					0.005***
					(0.001)
GP consultation in previous year					0.012***
					(0.001)
Specialist consultation in previous year					0.005***
					(0.001)
Socioeconomic controls	NO	YES	YES	YES	YES
McFadden's R^2	0.002	0.015	0.038	0.018	0.049
Log Likelihood	-7391.120	-7296.428	-7126.772	-7269.421	-7044.087
AIC	14788.241	14696.857	14371.544	14652.841	14198.173
BIC	14811.316	15096.826	14825.355	15091.269	14621.218
Observations	16183	16183	16183	16183	16183

Note: All models estimated using a GLM estimator with logit link function. Coefficients represent average partial effects. Robust standard errors in parentheses. Stars indicate statistical significance as follows: $^{***}p < 0.001$; $^{**}p < 0.01$; $^{*}p < 0.05$.

Table A8: Coefficient estimates of the regression for robustness check (one model)

	**Model 1**
Immigration status	
...First generation	0.008
	(0.007)
...Second generation	0.020
	(0.012)
Predictor sets	
Physical activity	
...Partially trained	-0.021
	(0.012)
...Trained	-0.006
	(0.011)
Pharmaceutical advice	0.004^{**}
	(0.001)
Alcohol consumption	-0.007
	(0.006)
Body mass index	0.001
	(0.001)
Smoking habits	
...Daily smoker	0.016
	(0.008)
...Occasional smoker	0.003
	(0.011)
GP consultation in previous year	0.009^{***}
	(0.001)
Health status	
...Bad	-0.070
	(0.056)
...Fair	-0.143^{**}
	(0.053)
...Good	-0.183^{***}
	(0.054)
...Very good	-0.214^{***}
	(0.054)
High cholesterol	-0.003
	(0.008)
Hypertension	0.013
	(0.009)
Specialist consultation in previous year	0.004^{***}
	(0.001)
Socioeconomic controls	YES
McFadden's R^2	0.057
Log Likelihood	-6985.852
AIC	14103.703
BIC	14611.356
Observations	16183

Note: All models estimated using a GLM estimator with logit link function. Coefficients represent average partial effects. Robust standard errors in parentheses. Stars indicate statistical significance as follows: $^{***}p < 0.001$; $^{**}p < 0.01$; $^{*}p < 0.05$.

Table A9: Coefficient estimates of the effect of immigration status on ED use estimated with OLS assuming a linear probability model

	**(1) Unadjusted**	**(2) Main**	**(3) Health Status**	**(4) Health Behaviors**	**(5) Healthcare Use**
Immigration status					
First generation	0.025***	0.007	0.007	0.006	0.010
	(0.004)	(0.006)	(0.006)	(0.006)	(0.007)
Second generation	0.045***	0.028**	0.027*	0.028*	0.024
	(0.007)	(0.011)	(0.011)	(0.011)	(0.012)
Predictor sets					
Health status					
...Bad			-0.116**		
			(0.038)		
...Fair			-0.262***		
			(0.036)		
...Good			-0.341***		
			(0.035)		
...Very good			-0.388***		
			(0.036)		
Body mass index			0.001		
			(0.001)		
Hypertension			0.022**		
			(0.008)		
High cholesterol			0.005		
			(0.007)		
Alcohol consumption				-0.008	
				(0.005)	
Smoking habits					
...Occasional smoker				0.003	
				(0.010)	
...Daily smoker				0.016*	
				(0.007)	
Physical activity					
...Partially trained				-0.033**	
				(0.012)	
...Trained				-0.019	
				(0.011)	
Pharmaceutical advice					0.006***
					(0.002)
GP consultation					0.012***
					(0.001)
Specialist consultation					0.005***
					(0.001)
Socioeconomic controls	NO	YES	YES	YES	YES
R^2	0.002	0.038	0.039	0.039	0.058
Adj. R^2	0.002	0.036	0.037	0.036	0.054
Observations	42238	20584	19903	20265	16884

Note: All models estimated using a linear model with robust standard errors (HC1). Coefficients represent average partial effects. Robust standard errors in parentheses. Stars indicate statistical significance as follows: $^{***}p < 0.001$; $^{**}p < 0.01$; $^{*}p < 0.05$.
